# Medication adherence and utilization in patients with schizophrenia or bipolar disorder receiving aripiprazole, quetiapine, or ziprasidone at hospital discharge: A retrospective cohort study

**DOI:** 10.1186/1471-244X-12-99

**Published:** 2012-08-02

**Authors:** Ariel Berger, John Edelsberg, Kafi N Sanders, Jose Ma J Alvir, Marko A Mychaskiw, Gerry Oster

**Affiliations:** 1 Policy Analysis Inc. (PAI), Four Davis Court, Brookline, Massachusetts, 02445, USA; 2 Pfizer Inc., New York, NY, USA

**Keywords:** Schizophrenia, Bipolar disorder, Antipsychotic agents, Utilization, Healthcare costs

## Abstract

**Background:**

Schizophrenia and bipolar disorder are chronic debilitating disorders that are often treated with second-generation antipsychotic agents, such as aripiprazole, quetiapine, and ziprasidone. While patients who are hospitalized for schizophrenia and bipolar disorder often receive these agents at discharge, comparatively little information exists on subsequent patterns of pharmacotherapy.

**Methods:**

Using a database linking hospital admission records to health insurance claims, we identified all patients hospitalized for schizophrenia (ICD-9-CM diagnosis code 295.XX) or bipolar disorder (296.0, 296.1, 296.4-296.89) between January 1, 2001 and September 30, 2008 who received aripiprazole, quetiapine, or ziprasidone at discharge. Patients not continuously enrolled for 6 months before and after hospitalization (“pre-admission” and “follow-up”, respectively) were excluded. We examined patterns of use of these agents during follow-up, including adherence with treatment (using medication possession ratios [MPRs] and cumulative medication gaps [CMGs]) and therapy switching. Analyses were undertaken separately for patients with schizophrenia and bipolar disorder, respectively.

**Results:**

We identified a total of 43 patients with schizophrenia, and 84 patients with bipolar disorder. During the 6-month period following hospitalization, patients with schizophrenia received an average of 101 therapy-days with the second-generation antipsychotic agent prescribed at discharge; for patients with bipolar disorder, the corresponding value was 68 therapy-days. Mean MPR at 6 months was 55.1% for schizophrenia patients, and 37.3% for those with bipolar disorder; approximately one-quarter of patients switched to another agent over this period.

**Conclusions:**

Medication compliance is poor in patients with schizophrenia or bipolar disorder who initiate treatment with aripiprazole, quetiapine, or ziprasidone at hospital discharge.

## Background

Schizophrenia is a chronic, debilitating mental disorder that affects approximately 1% of all persons in the US [1]. Symptoms include -- but are not limited to--hallucinations, delusions, movement disorders, cognitive impairment, and lack of pleasure in everyday life. Onset of schizophrenia typically occurs before the age of 45 years; it is equally likely to affect men and women as well as members of all ethnic groups [2]. Antipsychotic drugs are the mainstay of treatment. Older medications, including haloperidol and chlorpromazine, often cause acute and chronic extrapyramidal side effects (e.g., rigidity, tremors, tardive dyskinesia). Second-generation (often termed “atypical”) antipsychotic drugs infrequently induce these side effects.

Bipolar disorder, which is similarly debilitating, has been estimated to affect about 1% of persons in the US [3]. It is characterized by dramatic mood swings—from manic states (e.g., increased energy, euphoric moods, poor judgment, provocative and aggressive behavior) to depressive states (e.g., persistent sad mood, feelings of guilt, loss of interest in activities once enjoyed), and then back again, typically with periods of normal mood in between. Long-term therapy with “mood stabilizers” (e.g., lithium, valproate, lamotrigine) is often used to control the disease. However, other medications, including second-generation antipsychotics (SGAs), are often used on a short-term basis to treat “breakthrough” episodes of mania or depression.

While SGAs generally are considered to be better tolerated than older agents, such as haloperidol and chlorpromazine [4-6], their side effects--including weight gain, increased serum prolactin levels, hyperglycemia, and diabetes--can lead to therapy discontinuation, resulting in an increased risk of relapse, higher utilization of inpatient services, and higher costs of care [7]. SGAs also have been associated with an increased risk of death in older patients with dementia [8]. (Patients taking clozapine are also at risk of agranulocytosis.) Results from a large randomized trial in patients with schizophrenia sponsored by the National Institute of Mental Health (Clinical Antipsychotic Trials of Intervention Effectiveness [CATIE]) [9] reported that “overall effectiveness” (as measured by discontinuation at 18 months) was similar between the first-generation antipsychotic, perphenazine, and three of the four SGAs that were studied (risperidone, quetiapine, ziprasidone) (the fourth SGA in CATIE, olanzapine, had a significantly lower rate of discontinuation [64% vs 75% for perphenazine], albeit still high in absolute terms).

In this study, we examine patterns of pharmacotherapy among patients who were hospitalized for schizophrenia or bipolar disorder and received aripiprazole, quetiapine, or ziprasidone at discharge. We focused attention on aripiprazole, quetiapine, or ziprasidone, as we believe these SGAs are relatively similar with respect to their adverse event profiles. Olanzapine and risperidone were excluded due to much higher reported rates of weight gain with the former, and extrapyramidal symptoms (EPS) with the latter [8].

## Methods

### Data source

Data for this study were obtained from a unique linkage between the Truven (formerly Thomson Reuters) MarketScan® Commercial Claims and Encounters Database (a health insurance claims database) and the Truven MarketScan® Hospital Drug Database (an admission-level database).

The MarketScan Commercial Claims and Encounters Database is comprised of paid institutional, provider, and retail pharmacy claims from a variety of private insurers, representing healthcare services provided to approximately 10 million persons annually in the US. This database contains information on patient demographics and eligibility, inpatient and outpatient diagnoses (in International Classification of Diseases, 9^th^ edition [ICD-9-CM] format), inpatient and outpatient procedures (in ICD-9-CM, Physician Current Procedural Terminology, 4^th^ edition [CPT-4], and Health Care Financing Administration Common Procedure Coding System [HCPCS] formats), drugs dispensed in an outpatient setting (using National Drug Codes) (NDC), and dates of service for all medical services and drugs. All data can be arrayed longitudinally to provide a detailed profile of the medical and pharmacy services used by each person over time.

The MarketScan Hospital Drug Database contains admission-level information extracted from hospital decision-support and/or cost-accounting systems on about 3 million discharges annually from approximately 150 US, short-term, general, acute-care hospitals. For each admission, the database includes (but is not limited to) information on: (1) patient demographics (age, gender); (2) principal and secondary diagnoses (in ICD-9-CM format); (3) principal and secondary procedures (in ICD-9-CM format); (4) primary payer; (5) length of stay; (6) day-of-stay data; (7) drug utilization; (8) discharge status and destination; (9) department charge detail; and (10) total inpatient charges.

All patient identifiers in both databases have been fully encrypted, and both databases are fully compliant with the Health Insurance Portability and Accountability Act of 1996 (HIPAA). Since both databases were fully de-identified and our research was retrospective, ethics approval was unnecessary and therefore was not sought.

Information from the two databases was linked prior to the analysis. This linkage permitted us to examine patient characteristics in the period prior to initial hospitalization, and also allowed for more complete ascertainment of the use of SGAs. Data for this study spanned the period January 1, 2001 through September 30, 2008 (“study period”).

### Study sample

Using the MarketScan claims database, two study cohorts were constituted. The first consisted of all patients with evidence of any hospital admission with a principal diagnosis of schizophrenia (ICD-9-CM diagnosis code 295.XX) during the study period (“schizophrenia cohort”); the second, all patients with evidence of any hospital admission for bipolar mania (296.0, 296.1, 296.4-296.89) during the same period (“bipolar cohort”). Information for these selected patients was then extracted from the Hospital Drug Database, and data from the two files were then merged. Patients in the healthcare claims database who could not be linked to the admission-level database were dropped from the study sample. Among all remaining patients, attention was focused on those who received oral ziprasidone, aripiprazole, or quetiapine on their day of hospital discharge or the immediately preceding one (i.e., as their discharge medication).

Patients not continuously enrolled for 6 months prior to and following their hospital admission (“pre-admission” and “follow-up”, respectively) were excluded. Attention was limited to the *first* “qualifying” hospitalization only if patients had more than one. We excluded patients if they: (1) received clozapine any time during the study period; (2) were enrolled in an insurance plan with a mental health “carve-out” (and thus were likely to have incomplete data on healthcare services received for the treatment of mental disorders); (3) were aged <18 years as of the date of hospital admission; (4) had any healthcare encounters with a diagnosis of epilepsy (ICD-9-CM diagnosis codes 345.1, 345.4, 345.5) anytime prior to the qualifying hospital admission (antiepileptics are sometimes used as mood stabilizers); or (5) had evidence of both schizophrenia and bipolar disorder during the study period.

All information from both data systems were then compiled for all patients in the study sample from the beginning of the 6-month pre-admission period to the end of the 6-month follow-up period.

### Measures and analyses

The demographic and clinical characteristics of study subjects were examined, including their age, sex, plan type, geographic region, and the prevalence of selected comorbidities (Table 1), based on information from the qualifying admission and the 6-month pre-admission period. Patients were assumed to have a particular comorbidity if they had either one or more hospitalizations, or two or more outpatient claims at least 30 days apart, during the pre-admission period with a corresponding diagnosis code. Levels of utilization of antipsychotics and other psychotropic medications during the 6-month pre-admission period also were examined, along with total healthcare costs. We also examined selected characteristics of the hospitals to which patients were admitted (e.g., number of beds, teaching status).

**Table 1:** Diagnoses (in ICD-9-CM format) used to identify comorbidities of interest

**Comorbidity**	**ICD-9-CM Diagnosis Codes**
Depressive disorders	311, 296.2X, 296.3X, 296.5X, 296.82, 300.4, 298.0, 309.0, 309.28, 309.1
Dementia	290.XX, 291.2X, 310.9X, 331.0
Anxiety disorders	300.XX, 301.XX, 309.21
Post-traumatic stress disorder (PTSD)	309.81, 308.XX
Insomnia	780.50, 780.51, 780.53
Other psychoses	297.XX-299.XX, 300.1X, 302.8X, 307.9X
Diabetes	250.XX
Bulimia nervosa	307.51
Impulse-control disorder	312.30
Chronic fatigue syndrome	780.71
Hypertension	401-405
Obesity	278.XX
Arthritis	715-716
Chronic obstructive pulmonary disease (COPD)	496
Cerebrovascular disease	430-438.XX
Coronary heart disease	410-414.XX
Dyslipidemia	272.4
Alcohol/drug abuse	303-305.XX
Suicide/self-harm attempts	300.9, 959.9, E950.X-E959.X

Patterns of use of study agents were examined during the follow-up period, including adherence and therapy switching. Adherence was examined using medication possession ratios (MPRs) and cumulative medication gaps (CMGs). MPR was calculated as the ratio of the total number of therapy-days supplied during follow-up (beginning with day of hospital discharge, and ending with last day of follow-up) to the total number of calendar days in follow-up, which was the same for each patient in the study sample (i.e., 183 days). (Therapy-days that extended beyond the last day of follow-up were truncated as of this date.) CMG was calculated as the ratio of the difference between the total number of days of follow-up minus the total number of therapy-days, to the total number of days of follow-up.

Therapy switching, which has been reported to be associated with an increased likelihood of emergency room encounters, hospital admissions, and higher healthcare costs [10,11], was defined as receipt of a “new” antipsychotic following initiation of discharge therapy (i.e., one not used during either the pre-admission period or the qualifying admission) *without* any evidence of receipt of the initial study agent ≥60 days following treatment initiation with the “new” one. The date of therapy switching was assumed to be the date of first receipt of the new antipsychotic.

Use of other psychotropic medications during follow-up was examined in terms of the numbers of patients with any prescriptions for such therapies as well as the numbers of prescriptions for—and therapy-days with—these medications. Medications of interest included: (1) other antipsychotics, including other SGAs (e.g., olanzapine, risperidone), and all others (e.g., flupenthixol, haloperidol, perphenzine, thioridazine); (2) mood stabilizers (e.g., lithium, antiepileptics); (3) antidepressants (including selective-serotonin reuptake inhibitors [SSRIs], serotonin-norepinephrine reuptake inhibitors [SNRIs], tricyclic antidepressants [TCAs], monoamine oxidase [MAO] inhibitors, mirtazpine, nefazodone, buproprion); and (4) sedatives/hypnotics (e.g., benzodiapines, amobarbital, zolpidem).

Simple descriptive statistics (e.g., frequency counts, percentages, means, medians, SD) were used to describe each measure. Kaplan-Meier methods were used to depict the incidence of therapy switching. Significance testing was not undertaken as there were no *a priori* hypotheses (i.e., all analyses are descriptive in nature). All analyses were done separately for the schizophrenia and bipolar cohort, respectively. All analyses were conducted using PC-SAS® v.9.1.3.

## Results

### Schizophrenia cohort

**Figure 1:**
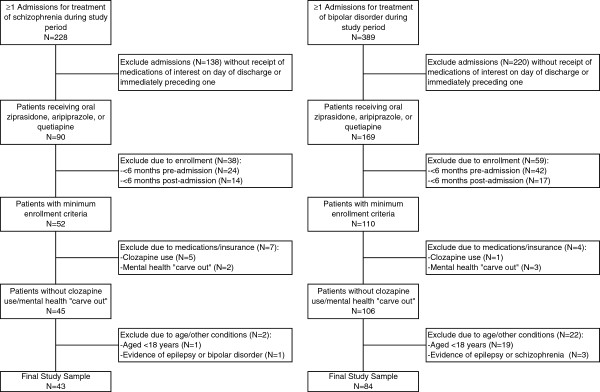
Sample selection criteria.

A total of 228 patients were identified with at least one admission for the treatment of schizophrenia during the study period; after applying all remaining study entry criteria, the total number of patients who qualified for entry into the study was 43 (Table 2, Figure 1). Mean (SD) age of patients with schizophrenia was 46.1 (13.0) years and 58.1% were women (Table 3). A total of 67.4% of patients had evidence of comorbid mental disorders, including depressive disorders (39.5%), other psychoses (25.6%) and alcohol/drug abuse (20.9%); 23.3% had evidence of diabetes. At hospital discharge, 30.2% of patients received aripiprazole; 34.9%, quetiapine; and 34.9%, ziprasidone. During initial hospitalization for schizophrenia, no patients received olanzapine, 3 (6.9%) patients received risperidone, and 9 (20.9%) patients received other antipsychotics, most often haloperidol (n = 7 [16.3%]). Mean (SD) duration of therapy with the three agents of interest (i.e., aripiprazole, quetiapine, ziprasidone) during initial hospitalization for schizophrenia was 7.8 (6.0) days; the mean (SD) cost was $6512 ($7666).

**Table 2:** Sample selection

**Criteria**	**Number of Patients in**	
	**Schizophrenia Cohort**	**Bipolar Disorder Cohort**
Total number of patients with ≥1 inpatient admissions for treatment of condition of interest* during study period** and	228	389
Received oral ziprasidone, ariprazole, or quetiapine on the day of discharge or immediately preceding one and	90	169
≥6 months enrollment prior to “qualifying” admission and	66	127
≥6 months enrollment subsequent to date of discharge of “qualifying” admission and	52	110
Had no evidence of receipt of clozapine at any time during study period and	47	109
Were not enrolled in insurance plan with mental health “carve out” and	45	106
Were aged ≥18 years as of date of qualifying hospital admission and	44	87
Had no evidence of epilepsy prior to qualifying admission and	44	85
Had no evidence of other condition of interest* during study period	43	84

*Either schizophrenia or bipolar disorder.

*For patients with multiple such admissions, only the first such admission will be examined; study period spans the period January 1, 2001 to September 30, 2008.

**Table 3:** Demographic and clinical characteristics, by study cohort*

**Characteristic**	**Schizophrenia Cohort**	**Bipolar Cohort**
	**(N = 43)**	**(N = 84)**
Mean (SD) age	46.1 (13.0)	44.5 (15.0)
Female	25 (58.1)	62 (73.8)
Payer type		
HMO	3 (7.0)	9 (10.7)
POS	21 (48.8)	48 (57.1)
PPO	5 (11.6)	12 (14.3)
Other/unknown	14 (32.6)	15 (17.9)
Comorbidities		
Mental disorders		
Depressive disorders	17 (39.5)	43 (51.2)
Dementia	0 (0.0)	0 (0.0)
Anxiety disorders	4 (9.3)	31 (36.9)
PTSD	0 (0.0)	0 (0.0)
Insomia	0 (0.0)	0 (0.0)
Other psychoses	11 (25.6)	19 (22.6)
Bulinia nervosa	0 (0.0)	0 (0.0)
Impulse-control disorder	0 (0.0)	0 (0.0)
Chronic fatigue syndrome	0 (0.0)	0 (0.0)
Alcohol/drug abuse	9 (20.9)	12 (14.3)
Suicide/self harm attempts	0 (0.0)	0 (0.0)
Any of above	29 (67.4)	69 (82.1)
All other		
Diabetes	10 (23.3)	13 (15.5)
Hypertension	9 (20.9)	21 (25.0)
Obesity	3 (7.0)	3 (3.6)
Arthritis	2 (4.7)	2 (2.4)
COPD	2 (4.7)	3 (3.6)
Cerebrovascular disease	1 (2.3)	1 (1.2)
Coronary heart disease	4 (9.3)	4 (4.8)
Dyspilidemia	0 (0.0)	1 (1.2)
Characteristics of admitting hospital**		
Number of beds		
≤200	4 (9.3)	8 (9.5)
201-300	5 (11.6)	31 (36.9)
>300	32 (74.4)	45 (53.6)
Non-teaching facilities	37 (86.0)	78 (92.9)
Receipt during pre-admission period of		
Antipsychotics		
Atypicals	40 (93.0)	65 (77.4)
Others	17 (39.5)	12 (14.3)
All of the above	41 (95.3)	66 (78.6)
All other psychotropic medications	33 (76.7)	78 (92.9)
Total costs, $		
Mean (SD) total pre-admission costs, $	13,696 (9,517)	13,747 (11,236)

*Unless otherwise indicated, all values are number of patients (%).

HMO: Health-maintenance organization; POS: Point of service; PPO: Preferred-provider organization; PTSD: Post-traumatic stress disorder; COPD: Chronic obstructive pulmonary disorder.

**Figure 2:**
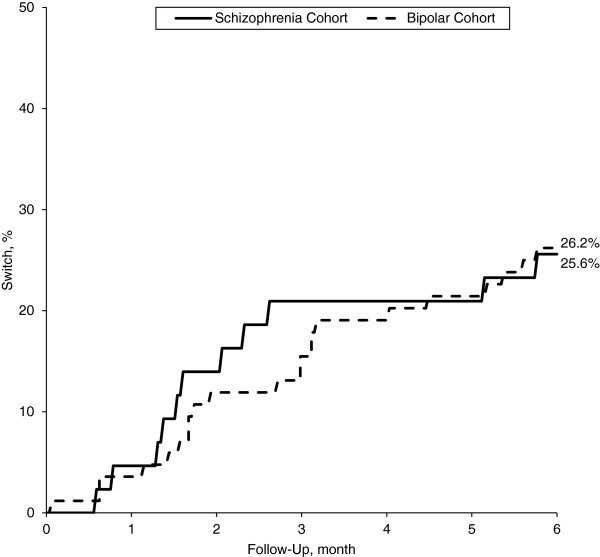
Incidence of therapy switching during follow-up, by study cohort.

During the 6-month follow-up period, study subjects averaged 7.2 (7.4) prescriptions for the study agent prescribed at discharge, spanning 100.9 (69.0) therapy-days; mean MPR and CMG were 55.1% (37.7%) and 44.9% (37.7%), respectively (Table 4). Eighty-four percent of patients filled at least one prescription for the study agent prescribed at discharge. Twenty-six percent of patients had switched to another antipsychotic agent by 6 months; most instances of therapy switching occurred within 3 months of discharge from the qualifying hospitalization (Figure 2).

**Table 4:** Magnitude of use of initial therapy during follow-up, by study cohort

	**Schizophrenia Cohort**	**Bipolar Cohort**
	**(N = 43)**	**(N = 84)**
Prescriptions		
Mean (SD)	7.2 (7.4)	4.2 (3.5)
Median (IQR)	5 (3, 8)	3 (2, 6)
Therapy-days		
Mean (SD)	100.9 (69.0)	68.3 (60.5)
Median (IQR)	121 (31,166)	46 (31,115)
MPR		
<10%	8 (18.6)	20 (23.8)
10-20%	6 (14.0)	22 (26.2)
21-30%	1 (2.3)	0 (0.0)
31-40%	2 (4.7)	7 (8.3)
41-50%	0 (0.0)	11 (13.1)
51-60%	2 (4.7)	2 (2.4)
61-70%	4 (9.3)	6 (7.1)
71-80%	4 (9.3)	3 (3.6)
81-90%	6 (14.0)	3 (3.6)
91-100%	10 (23.3)	10 (11.9)
Mean (SD)	55.1 (37.7)	37.3 (33.1)
CMG		
<10%	11 (25.6)	10 (11.9)
10-20%	8 (18.6)	4 (4.8)
21-30%	1 (2.3)	2 (2.4)
31-40%	4 (9.3)	6 (7.1)
41-50%	2 (4.7)	13 (15.5)
51-60%	0 (0.0)	1 (1.2)
61-70%	2 (4.7)	6 (7.1)
71-80%	1 (2.3)	0 (0.0)
81-90%	6 (14.0)	22 (26.2)
91-100%	8 (18.6)	20 (23.8)
Mean (SD)	44.9 (37.7)	62.7 (33.1)

*Unless otherwise indicated, all values are number of patients (%).

MPR: Medication possession ratios; CMG: Cumulative medication gap.

Use of psychotropic medications during follow-up was common. Patients averaged 8.7 (8.5), 2.1 (4.8), 5.2 (6.5), 4.0 (4.6), and 4.6 (9.4) pharmacy dispenses for SGAs (including study agents), other antipsychotics, mood stabilizers, antidepressants, and sedatives/hypnotics, respectively; corresponding values for therapy-days were 162.9 (105.7), 31.0 (80.1), 114.8 (132.1), 86.6 (93.1), and 27.7 (66.3), respectively.

### Bipolar disorder cohort

A total of 389 patients were identified who had at least one admission for the treatment of bipolar disorder during the study period; after applying all remaining study entry criteria, the total number of patients who qualified for entry into the study was 84 (Table 2, Figure 1). Mean (SD) age of patients with bipolar disorder was 44.5 (15.0) years, and 73.8% were women (Table 3). Most patients (82.1%) had evidence of other mental disorders, including depressive disorders (51.2%), anxiety disorders (36.9%), other psychoses (22.6%), and alcohol/drug abuse (14.3%); 15.5% had evidence of diabetes. At hospital discharge, 15.5% of patients received aripiprazole; 56.0%, quetiapine; and 28.6%, ziprasidone. During the initial hospitalization for bipolar disorder, no patients received olanzapine, 6 (7.1%) patients received risperidone, and 11 (13.1%) patients received other antipsychotics, most often mesoridazine (n = 4 [4.8%]) or haloperidol (n = 3 [3.6%]). Mean (SD) duration of therapy with the three agents of interest (i.e., aripiprazole, quetiapine, ziprasidone) during initial hospitalization for bipolar disorder was 4.6 (4.3) days; the mean (SD) cost was $6663 ($8900).

During the 6-month period of follow-up, these patients averaged 4.2 (3.5) prescriptions for the study agent prescribed at discharge, spanning 68.3 (60.5) therapy-days; mean MPR and CMG were 37.3% (33.1%) and 62.7% (33.1%), respectively (Table 4). Seventy-four percent of patients filled at least one prescription for the study agent prescribed at discharge. Twenty-six percent of patients had switched to another antipsychotic agent by 6 months; most instances of therapy-switching occurred within 3 months of discharge from the qualifying hospitalization (Figure 2).

Use of psychotropic medications during follow-up was common. Patients averaged 4.6 (2.8), 0.7 (1.6), 4.4 (3.6), 2.0 (2.7), and 2.0 (2.9) pharmacy dispenses for SGAs (including study agents), other antipsychotics, mood stabilizers, antidepressants, and sedatives/hypnotics, respectively; corresponding values for therapy-days were 111.1 (103.8), 16.0 (47.2), 118.3 (121.0), 56.2 (75.8), and 47.6 (96.1), respectively.

## Discussion

In our study, patients with schizophrenia or bipolar disorder who were recently discharged from the hospital were found to have poor adherence over time with aripiprazole, quetiapine, and ziprasidone. Those with schizophrenia received medication sufficient to cover only about 55% of their follow-up days over 6 months (mean CMG), while those with bipolar disorder received medication sufficient to cover only about 37% of their follow-up days. Approximately one in four patients had evidence of therapy switching during the 6-month follow-up period.

Noncompliance is an important predictor of hospitalization risk. Following inpatient treatment and discharge from the community, many patients become poorly compliant with therapy [12,13]. Several reasons for poor compliance have been hypothesized, including disease symptoms (e.g., grandiosity, paranoia, problems with accurate recall), treatment-emergent side effects, substance abuse, lack of support systems to encourage medication compliance, psychostressors, and poor patient-provider relationships [14,15]. Previous reports suggest that more than one-half of all patients with schizophrenia become noncompliant with their medication regimen within one month of discharge from hospital [15]; by 2 years following discharge, about 75% are noncompliant [15-17]. Among patients with bipolar disorder, compliance is also low. In one retrospective analysis of healthcare claims data, Lage and Hassan report that 62% of patients with bipolar disorder newly started on antipsychotics had MPRs ≤50%, and that mean MPR was 41.7% [18]. Rates of medication adherence were low in our study too.

We limited our attention to aripiprazole, quetiapine, or ziprasidone, as we believed these three SGAs to be relatively similar with respect to their adverse event profiles. Olanzapine and risperidone were excluded due to much higher reported rates of weight gain with the former, and extrapyramidal symptoms (EPS) with the latter [8]. Our results suggest that adherence is not substantially better with these agents than with other SGAs (e.g., rates of discontinuation in the CATIE study ranged from 64% [olanzapine] to 82% [quetiapine]) [19]. We note, however, that our study sample was small, which limits the generalizability of our findings. Further study is needed to better understand patterns of “real-world” adherence among patients prescribed SGAs upon discharge from hospital for schizophrenia and bipolar disorder, respectively.

This study has a few key limitations. For one, our sample size was small, principally because the study dataset was created by linking information from a health insurance claims database with data from a large inpatient data warehouse. Compounding this limitation was our decision to limit the study sample to patients with at least 6 months of eligibility for medical and drug benefits before and after the “qualifying” hospitalization, and to exclude patients with evidence of both schizophrenia and bipolar mania. Accordingly, the generalizability of our findings to all patients hospitalized for schizophrenia or bipolar disorder who are subsequently discharged on SGAs is unknown. On a related note, while prior research in both schizophrenia and bipolar disorder has established an inverse correlation between levels of adherence with antipsychotic medications and risk of relapse [20-32], the relatively small number of patients in our study precluded an examination of this question.

Second, the healthcare claims database—the source for most of the information on patterns of utilization of study agents during follow-up—only allows the identification of prescription drugs dispensed by retail pharmacies (i.e., filled prescriptions and their associated therapy-days). We could not ascertain whether medications that were dispensed were actually taken. Thus, our estimates may represent an upper bound for the amount of medication actually taken. On the other hand, it should be noted that patients are sometimes discharged from hospital with a small supply (e.g., 3 days) of medication to insure continued use until they are able to fill a prescription at a retail pharmacy. Unfortunately, the database does not contain information on professional samples dispensed at hospital discharge. In our study, 39%, 56%, and 69% of patients in the schizophrenia cohort filled their first prescription for study agents within one, six, and 14 days of discharge from their qualifying hospitalization, respectively; corresponding values for patients in the bipolar cohort were 61%, 81%, and 82%, respectively. It is reasonable to assume that most of these patients were intent on continuing with the study agent received at discharge. Accordingly, our reliance on therapy-days as noted on paid claims from retail pharmacies likely understates the amount of medication actually taken. Further study is needed to better understand the degree to which patients with schizophrenia and/or bipolar disorder adhere to discharge therapy.

Finally, as with all database studies, there may be errors of omission and commission in coding. However, the case-finding algorithm (i.e., hospitalization with a principal diagnosis of schizophrenia or bipolar disorder, and initiation of SGA treatment at discharge) used in this analysis likely minimized the inclusion of patients who did not have schizophrenia or bipolar disorder therefore, the specificity of this algorithm is likely high.

## Conclusions

In conclusion, this study suggests that adherence is a substantial problem in patients hospitalized for schizophrenia and bipolar disorder who are discharged on second-generation antipsychotics. Further research is needed to better understand the reason(s) for these poor levels of adherence among this group of high-risk patients, and the degree to which nonadherence may be associated with higher levels of healthcare utilization and costs following hospital discharge, including rehospitalization.

## Competing interests

Mr. Berger, Dr. Edelsberg, and Dr. Oster are employed by Policy Analysis Inc., an independent contract research organization who were paid consultants to Pfizer in connection with the development of this manuscript and previous and ongoing engagements with Pfizer Inc. as well as other pharmaceutical manufacturers. Ms. Sanders, Dr. Alvir, and Dr. Mychaskiw are employed by Pfizer Inc.

## Authors’ contributions

All authors reviewed and contributed to the study research plan, interpretation of the data, and the study manuscript; data management, processing, and analyses were conducted by AB, JE, and GO. All authors read and approved the final manuscript.

## Disclosures

Kafi N. Sanders, Jose Ma. J. Alvir and Marko A. Mychaskiw are all full-time employees of Pfizer, Inc.

## Financial support

Funding for this research was provided by Pfizer, Inc., New York, NY.

## Pre-publication history

The pre-publication history for this paper can be accessed here:

http://www.biomedcentral.com/1471-244X/12/99/prepub
